# Effects of dietary probiotic, liquid feed and nutritional concentration on the growth performance, nutrient digestibility and fecal score of weaning piglets

**DOI:** 10.5713/ajas.19.0473

**Published:** 2019-11-12

**Authors:** Song Zhang, Dong Huy Yoo, Xiang Ao, In Ho Kim

**Affiliations:** 1 Department of Animal Resource and Science, Dankook University, Cheonan 31116, Korea; 2 Kemin Industries (China) Co., Ltd. Sanzao, Zhuhai 519040, China; 3 All The Best Co., Ltd. Seoul 05399, Korea

**Keywords:** Dietary Probiotic Blend, Liquid Feeding Program, Nutrition Levels, Growth Performance, Fecal Score, Nutrient Digestibility, Weaning Pigs

## Abstract

**Objective:**

This study was conducted to investigate the effects of dietary probiotic blend and liquid feed program at different nutritional densities on growth performance, nutrient digestibility, fecal score of weaning piglets.

**Methods:**

A total of 120 weaning pigs with an initial body weight of 7.05±0.93 kg per pig (21 days of age) were randomly allocated into 1 of the following 8 dietary treatments (3 replicates per treatment with 5 pigs per replicate) in a 2×2×2 factorial arrangement (nutrition levels: apparent metabolic energy [AME] = 3,500 kcal/kg, crude protein [CP] = 20% vs AME = 3,400 kcal/kg, CP = 19.42%; feed types:dry vs wet; probiotics levels: 0 mg/kg vs 300 mg/kg).

**Results:**

During d 5 to d 15, greater average daily gain (ADG) and average daily feed intake (ADFI) (p<0.05) were observed in probiotics treatments. During d 15 to d 25, gain:feed (G:F) ratio (p<0.05) were significantly improved in probiotics, wet feed and high nutrition diet. Moreover, two interactions i) between nutrition levels and feed types, and ii) between nutrition levels and probiotics were found in G:F ratio. Furthermore, there was a significant positive interaction on G:F among those 3 factors (p<0.05). Overall, increasing ADG, ADFI, and G:F ratio were detected in probiotics treatment significantly (p<0.05). Besides, an obvious reduction on fecal score was observed in probiotics treatment from d 0 to d 5 (p<0.05). There was an interactive effect on fecal score between feed types and nutrition concentrations from d 5 to d 25 (p<0.05).

**Conclusion:**

These results indicated that probiotics supplementation could benefit growth performance and reduce the frequency of watery feces. Besides, wet feed program (feed:water = 1:1.25) could improve the G:F. The effect of liquid feed or probiotic could be influenced by dietary nutrition density in weaned piglets. An increased value of G:F was obtained when wet feeding a high nutrition diet (100 kcal higher than NRC 2012 recommendations) was supplemented with probiotics for 15 to 25 days.

## INTRODUCTION

Gastrointestinal disturbances immediately post weaning can cause heavy economic loss in the pig industry. The weaning transition is a complex period during which the piglets have to cope with abrupt separation from their mothers and adapt to new environment where they are mixed with other litters. In addition, their diet will be switched from highly-digestible (liquid) milk to a less digestible and more-complex solid feed during this transition. Weaned at an early age (21 to 35 d) in intensive production systems has probably exacerbated the level of general stress in these immature animals [1].

In the past decade, various nutritional methods or solutions to minimize the weaning losses have been tested, some of which have been widely implemented in practice. Probiotics, which is a modulator to increase many active behaviors, plays an important role in gut-brain axis regulation [2]. Many references demonstrate different probiotics capacities can enhance productivity in weaning piglets and increase gain:feed (G:F) ratio [3], average daily gain (ADG) [4] and nutrient digestibility [5]. Direct action of the probiotics can achieve a higher bioavailability of feed nutrients, indirect gut health modulation (relieving weaning stress, preventing diarrhea, improving the intestinal microbiota profile, etc.) or perhaps a combination of both may be involved [6]. Liquid feed diets have been widely used in western and southern Europe for 20 years, especially in France and Italy [7]. There has been an increase in the use of fermented liquid and liquid feed in the European Union (EU) since the ban on the use of antibiotics as growth promoters in pig feed [8]. Piglets grew faster on the liquid diet due to a higher feed intake (FI), a better transition from milk feeding and lower risk of dehydration [9]. With the advancement of additives and feeding technology, probiotics and liquid feeding would be used in most swine farms. However, what nutritionists always focus on is the formulation designs. Various nutrition levels and the liquid feed program might influence the effects of additives in pigs. Therefore, we hypothesized there might be an interaction among nutrition levels, the liquid feeding program and probiotics. However, no research has been carried out to determine the interaction among probiotics, the liquid feeding program and nutrition designs. Consequently, the objective of the study is to determine effects of dietary probiotic blend and liquid feed program at different nutritional densities on growth performance, nutrient digestibility, fecal score in weaning piglets.

## MATERIALS AND METHODS

The protocols used for the current experiment were approved by the Animal Care and Use Committee of Dankook University, Korea.

### Animal, diet, experimental design

A total of 120 weaning pigs (21 days of age) with an initial body weight (BW) of 7.05±0.93 kg per pig were randomly allocated into 1 of 8 dietary treatments (3 replicates per treatment with 5 pigs per replicate) in a 2×2×2 factorial arrangement with 2 levels of nutrition density apparent metabolic energy (AME) = 14.63 kJ/kg or 3,500 kcal/kg, CP = 20% vs AME = 14.23 kJ/kg or 3,400 kcal/kg, CP = 19.42%), 2 types of feed (dry vs wet), the liquid feed was prepared by mixing meal and water in a 1:2.5 ratio. The 2 levels of probiotics (0 mg/kg vs 300 mg/kg) were provided by Daehan feed mill company (*Bacillus subtilis* 2×10^8^ colony-forming unit [CFU]/g, *Enterococcus faecium* 2×10^8^ CFU/g). The 8 experimental diets were: TRT1, high nutrition × dry type without probiotics; TRT2, high nutrition × dry type with probiotics; TRT3, high nutrition × wet type without probiotics; TRT4, high nutrition × wet type with probiotics; TRT5, low nutrition × dry type without probiotics; TRT6, low nutrition × dry type with probiotics; TRT7, low nutrition × wet type without probiotics; TRT8, low nutrition × wet type with probiotics. This research was divided into three periods: Phase 1, d 0 to 5; Phase 2, d 5 to 15; Phase 3, d 15 to 25. All diets were formulated to contain approximately equal amounts of essential amino acids and the amino acid was on standard ileal digestibility basis. Ca, absorbable P, and Na, based on the analytical data from the feedstuffs. Different nutrition concentration diets meet or exceed the requirements suggested by the National Research Council (NRC [10]) (Table 1).

Chromic oxide (Cr_2_O_3_) was added to each diet at 0.2% as an indigestible marker to evaluate the nutrient digestibility on d 5, d 15, and d 25. Pigs were housed in an environmentally controlled nursery facility with slatted plastic flooring and a mechanical ventilation system. The temperature of the room was maintained at approximately 30°C for the first week of the experiment, after which it was reduced by 1°C per week over the next 4 weeks. Each pen (1.8 × 1.0 m) was equipped with a self-feeder and a nipple waterer to allow *ad libitum* access to feed and water throughout the experimental period.

### Sampling and measurements

The individual pig BW and feed consumption from each pen was monitored to calculate the ADG, average daily feed intake (ADFI) and G:F ratio on 0, d 5, d 15, and d 25. Fecal samples were collected from two pigs in each pen (1 gilt and 1 barrow) on the d 5, d 15, and d 25 of the experiment to determine the apparent total tract digestibility of dry matter (DM), N, and energy.

Feed samples were dried at 70°C for 72 h, after which they were ground to pass through a 1-mm screen. The DM and N concentrations were determined according to the AOAC [11]. Representative samples of each ingredient were hydrolyzed for 23 h at 110 1°C with or without performic acid oxidation for Cys and Met, and other AA, respectively, and AA was separated by ion exchange chromatography and quantified by photometric detection after ninhydrin reaction European Commission [12].

The fecal score was determined by the average value of five pigs of each pen by using a 5-grade score system [13]. The standard of this system is as following: 1 = hard, dry pellets in a small, hard mass; 2 = hard, formed stool that remains firm and soft; 3 = soft, formed and moist stool that retains its shape; 4 = soft, unformed stool that assumes the shape of the container; 5 = watery, liquid stool that can be poured. Scores were recorded on a pen basis following observations of individual pigs and signs of stool consistency in the pen.

### Statistical analyses

Data were analyzed as a completely randomized block design, with a 2×2×2 factorial arrangement, using general linear model procedure [14]. Variability in the data was expressed as the pooled standard error of mean, and p<0.05 was considered statistically significant, whereas p<0.10 was considered a tendency.

## RESULTS

### Growth performance and nutrient digestibility of dry matter, energy and nitrogen

The results of growth performance and nutrient digestibility of DM, energy and nitrogen are presented in Table 2, 3, respectively. In the phase 2, ADG and ADFI were greater p< 0.05) in probiotics treatments comparing to the non-probiotics treatments. In the phase 3, probiotics, wet feed and high nutrition diet significantly improved G:F (p<0.05). Besides, those pigs fed high nutrition diet appeared to have greater ADG and G:F than that fed low nutrition diet (p<0.05). Moreover, the interactive influence on G:F appeared not only between nutrition levels and feed types but also between nutrition levels and probiotics (p<0.05). Interestingly, there was a significant probiotics × feed type × nutrition density interaction on G:F (p<0.05). Piglets fed the diet containing probiotics had increased ADG, ADFI, and G:F comparing to that receiving the diet without probiotics (p<0.05). Besides, high nutrition diet significantly raised ADG and ADFI (p<0.05). No interactive response was found through the entire experiments (p>0.05). There was no difference in nutrient digestibility of DM, energy and nitrogen as well (p>0.05).

### Fecal score

The results of fecal score are appeared in Table 4. An obvious reduction on fecal score was observed in probiotics treatment from d 0 to d 5 (p<0.05). There was an interactive effect on fecal score between feed types and nutrition concentrations from d 5 to d 25 (p<0.05).

## DISCUSSION

The results of this study showed that dietary probiotics blend can increase the ADG, ADFI, and G:F ratio throughout the trial. And the results are accordance with the previous paper of our laboratory [15], which evidenced positive effects on growth performance in the overall period associated with the inclusion of multi-strain probiotics (*B. licheniformis* and *B. subtilis*) in the diets. Generally, probiotics or probiotics mixtures could improve ADG [16–18] and increase G:F ratio [19, 20] in post-weaned piglets. However, the influence of probiotics on ADFI is inconsistent. In agreement with our results, Nguyen et al [21] documented that increasing the inclusion of the probiotics mixture (*Bacillus coagulans*, *B. licheniformis*, *B. subtilis*, and *Clostridium butyricum*) levels in the diets linearly increased the ADG and ADFI for day 0 to 7 as well as ADG for day 8 to 21. However, Zhao et al [22] believed probiotics (*Lactobacillus reuteri* and *Lactobacillus plantarum*) blend could not affect ADFI. Different probiotics strains and concentration of probiotics might be an important factor affecting the ADFI. Especially, since probiotics also live on nutrient, they might compete with host for nutrients in the diet or make hosts require more feed.

The key factor underlying the poor postweaning performance is the immediate reduction in FI due to the abrupt transition from liquid milk to less digestible feeds [23,24], therefore, sufficient FI is a big challenge for subsequent growth performance. Previous studies indicated liquid feeding reconstituted to 13% DM and fed via liquid feeders in the early weaning period improves FI which resulted in greater body weight gain [25,26]. Whereas, this study showed liquid feed which were reconstituted to 25% DM only enhanced G:F ratio. The difference might result from the different DM. Geary et al [27] reported DM content in the range of 255 to 149 g/kg had no significant effect on DM intake post-weaning 4 weeks. Similarity, when Yang et al [28] fed the piglet liquid feed in a ratio of 1:2 from d 0 to d 28, no difference was found in ADFI, but there was an enhanced G:F. There are three possible reasons for this result. Firstly, water plays a crucial role in the muscle growth, which is a major part of the composition of organ and tissues, so enough water intake could be a reason for better G:F ratio. Secondly, a better transition from milk feeding can reduce the weaning stress and lead to a better G:F ratio. Thirdly, comparing with dry feed treatment, a lower fecal score and a tendency of better energy digestibility in wet feed group presented in this study also contributed to the improved G:F ratio.

Probiotics and nutrition density showed an interactive relationship in G:F ratio, which meant probiotics improved G:F ratio more dramatically in the high-nutrition diet. Similarly, our previous studies of Meng et al [29] and Yan et al [30] reported supplementation probiotics in high nutrition diets raised nutrient digestibility and reduced fecal gas emissions in growing pigs. And they believed that the interactive effect could be the increased microflora balance, which led to a better metabolism and transformation of feed into body mass. In our viewpoint, piglets fed relatively higher nutrition diet are more likely to suffer nutritional diarrhea, which results from that indigestible substrate inducing an explosive growth of bacteria and a disturbance of the colonization resistance [31]. Normally transient *Escherichia coli* strains in the gut [32] can multiply and attach. This study confirmed that the reason for positive effect in relatively high nutrition diet might be that probiotics play a role in balancing gut microflora, benefitting intestinal integrity to relieve intestinal stress under high nutrition [33]. However, Lan et al [34] whose paper reported the beneficial effects of probiotics complex supplementation on ADFI is more dramatic with low nutrient density (3,850 kcal/kg vs 4,000 kcal/kg), believed pigs were able to get same energy by increasing FI when low energy diets were provided. The difference interaction between nutrition level and probiotics might be caused by the actual energy in those trial diets and the different growth stages. There was a positive fecal score interaction between high nutrition diet and liquid feed. Relatively, the G:F ratio was improved more dramatically with liquid feed in the high-nutrition diet. Those two results might imply that comparing to NRC [10] nutrition recommendation, a higher nutrition formula should be considered when liquid feed is fed to piglets. Interestingly, there was an interaction on G:F among nutrition levels, probiotics and liquid feed in phase 3. Therefore, supplementation probiotics into liquid diet at high nutrition might be a whole solution to improve growth performance and health status in post-weaning pigs. When piglets are fed in different feeding programs or at various nutrition designs, additives applications should be considered specifically. However, the interaction could not be found in overall growth performance.

## CONCLUSION

These results indicated that probiotics in a supplementation diet could benefit growth performance (ADG, ADFI, and G:F) and reduce the frequency of watery feces. Besides, a wet feed program (feed:water = 1:1.25) could improve the G:F. Because there were two positive interactions: one between liquid program and nutrition density, the other between supplementation probiotics and nutrition density, the effect of liquid feed or probiotic could be influenced by dietary nutrition density in weaned piglets. An increasing value of G:F was obtained when wet feeding a high nutrition diet (100 kcal higher than NRC [10] recommendations) was supplemented with probiotics for 15 to 25 days.

## Figures and Tables

**Table 1:** Diet compositions (as fed basis)

Item	High nutrition diet	Low nutrition diet
Yellow corn	33.32	36.95
Extruded corn	20.00	20.00
Whey powder (78%)	7.00	7.00
Soybean meal (46%)	8.28	5.98
Fermented soybean meal	5.00	5.00
Extruded soybean meal	5.00	5.00
Skimmed milk powder	7.00	7.00
Fish meal	3.00	3.00
Sugar	2.00	2.00
Glucose	2.00	2.00
SDPP	3.00	3.00
Soy oil	1.33	0.00
Limestone	0.55	0.55
MCP	0.68	0.72
Salt	0.10	0.10
Lysine-HCl (98.5%)	0.44	0.42
DL-methionine (99%)	0.30	0.29
L-threonine (98.5%)	0.20	0.19
L-tryptophan (10%)	0.30	0.30
Choline (50%)	0.10	0.10
Vitamin premix1)	0.20	0.20
Mineral premix2)	0.20	0.20
Total	100.0	100.0
Calculated composition3) (%)		
CP	20.00	19.42
Crude fat	4.70	3.42
Ash	5.49	6.05
AME (kcal/kg)	3500	3400
Ca	0.7	0.7
AP	0.5	0.5
Lys	1.58	1.55
Apparent ileal digestible amino acid		
SID-Lys	1.48	1.45
ME/CP	175	175
CP/SID-Lys	13.79	13.79
SID-TSAA/SID-Lys	0.60	0.60
SID-Thr/SID-Lys	0.62	0.62
SID-Trp/SID-Lys	0.17	0.17

SDPP, spray-dried porcine plasma; MCP, monocalcium phosphate; AME, apparent metabolic energy; SID, standard ileal digestibility; CP, crude protein; TSAA, total sulfur amino acid.

1) Supplied per kg diet: 4,000 IU vitamin A, 800 IU vitamin D_3_, 171 IU vitamin E, 2 mg vitamin K, 4 mg vitamin B_2_, 1 mg vitamin B_6_, 16 μg vitamin B_12_, 11 mg pantothenic acid, 20 mg niacin and 0.08 mg biotin.

2) Supplied per kg diet: 220 mg Cu, 175 mg Fe, 191 mg Zn, 89 mg Mn, 0.3 mg I, 0.5 mg Co and 0.4 mg Se.

3) Calculated values.

**Table 2:** Effects of feeding program on growth performance in weaning pigs

Items	TRT11)	TRT21)	TRT31)	TRT41)	TRT51)	TRT61)	TRT71)	TRT81)	SEM	p-value2)						
		
	High nutrition density				Low nutrition density					Probiotics	Feed type	Nutrition density	Probiotics × feed type	Feed type × nutrition density	Probiotics × nutrition density	Interaction
	
	Dry type		Wet type		Dry type		Wet type									
	
	NC	Probiotics	NC	Probiotics	NC	Probiotics	NC	Probiotics								
Body weight (kg)																
Initial	7.08	7.08	7.07	7.05	7.04	7.04	7.02	7.02	0.61	0.930	0.963	0.985	0.997	0.991	0.994	0.994
Phase13)	8.09	8.16	8.13	8.16	8.16	8.09	8.03	8.11	0.63	0.940	0.966	0.951	0.928	0.957	0.963	0.913
Phase23)	10.75	10.95	10.82	11.22	10.12	10.35	10.20	10.54	0.69	0.216	0.755	0.557	0.975	0.874	0.988	0.964
Phase33)	13.56	15.15	14.38	15.84	12.79	14.11	12.93	14.31	1.02	0.118	0.535	0.064	0.690	0.982	0.906	0.951
Phase1																
ADG (g)	201	216	213	221	224	211	201	218	18	0.970	0.980	0.608	0.532	0.634	0.716	0.485
ADFI (g)	212	217	239	237	234	233	219	239	20	0.739	0.515	0.697	0.336	0.818	0.765	0.631
G:F	0.968	0.998	0.889	0.930	0.960	0.907	0.917	0.916	0.032	0.366	0.063	0.864	0.234	0.495	0.197	0.663
Phase2																
ADG (g)	266	279	269	307	196	225	217	243	20	<0.001	0.248	0.083	0.891	0.723	0.945	0.616
ADFI (g)	332	349	314	424	266	315	290	329	24	0.005	0.183	0.106	0.768	0.238	0.560	0.145
G:F	0.800	0.802	0.859	0.723	0.738	0.727	0.751	0.738	0.046	0.099	0.986	0.242	0.739	0.300	0.415	0.313
Phase3																
ADG (g)	281	419	356	461	267	376	273	376	42	0.078	0.323	0.002	0.366	0.751	0.805	0.826
ADFI (g)	418	623	529	669	452	551	403	565	61	0.141	0.486	0.003	0.283	0.986	0.634	0.470
G:F	0.668	0.674	0.672	0.690	0.587	0.684	0.676	0.666	0.010	0.005	0.006	0.001	0.093	0.004	0.042	<0.001
Overall																
ADG (g)	259	323	292	352	230	283	236	291	21	0.007	0.220	0.002	0.448	0.978	0.815	0.918
ADFI (g)	342	432	385	484	334	393	321	405	28	0.031	0.256	<0.001	0.249	0.666	0.569	0.851
G:F	0.756	0.747	0.763	0.726	0.687	0.720	0.736	0.719	0.016	0.011	0.482	0.509	0.196	0.105	0.182	0.625

SEM, pooled standard error of means; NC, negative control; ADG, average daily gain; ADFI, average daily feed intake; G:F, gain:feed.

1) TRT1, high nutrition density feed × dry type × none; TRT2, high nutrition density feed × dry type × probiotics; TRT3, high nutrition density feed × wet type × none; TRT4, high nutrition density feed × wet type × probiotics; TRT5, low nutrition density feed × dry type × none; TRT6, low nutrition density feed × dry type × probiotics; TRT7, low nutrition density feed × wet type × none; TRT8, low nutrition density feed × wet type × probiotics.

2) p<0.05 was considered statistically significant, whereas p<0.10 was considered a tendency.

3) Phase 1, 0 to 5days; Phase 2, 5 to 15 days; Phase 3, 15 to 25 days.

**Table 3:** Effects of feeding program on digestibility in weaning pigs

Items (%)	TRT11)	TRT21)	TRT31)	TRT41)	TRT51)	TRT61)	TRT71)	TRT81)	SEM	p-value2)						
		
	High nutrition density				Low nutrition density					Probiotics	Feed type	Nutrition density	Probiotics × feed type	Feed type × nutrition density	Probiotics × nutrition density	Interaction
	
	Dry type		Wet type		Dry type		Wet type									
	
	NC	Probiotics	NC	Probiotics	NC	Probiotics	NC	Probiotics								
5 d																
Dry matter	83.31	84.21	84.38	84.77	84.21	84.25	83.50	83.97	0.53	0.245	0.315	0.341	0.637	0.276	0.168	0.750
Nitrogen	82.64	83.23	83.81	82.58	83.03	83.65	83.64	83.69	0.91	0.156	0.267	0.543	0.104	0.739	0.624	0.448
Energy	83.86	84.35	84.75	85.36	84.68	83.79	85.31	85.22	0.77	0.265	0.543	0.438	0.681	0.963	0.624	0.541
10 d																
Dry matter	84.50	84.93	83.51	85.06	84.44	83.79	85.04	83.89	0.95	0.854	0.453	0.631	0.360	0.357	0.400	0.509
Nitrogen	83.57	82.65	83.62	84.31	83.65	84.31	82.86	83.06	0.59	0.804	0.654	0.265	0.664	0.631	0.817	0.168
Energy	83.83	84.35	84.75	85.36	84.32	85.28	83.31	85.16	0.80	0.647	0.169	0.735	0.261	0.736	0.547	0.440
25 d																
Dry matter	84.34	85.94	85.08	86.89	84.32	84.78	85.43	84.66	0.69	0.122	0.174	0.116	0.725	0.599	0.060	0.458
Nitrogen	82.61	84.85	83.83	85.05	82.97	83.41	83.60	83.41	0.78	0.187	0.361	0.099	0.724	0.458	0.154	0.863
Energy	83.97	84.80	84.45	86.01	82.84	84.10	84.42	83.83	0.80	0.078	0.189	0.180	0.863	0.620	0.448	0.257

SEM, pooled Standard error of means; NC, negative control.

1) TRT1, high nutrition density feed × dry type × none; TRT2, high nutrition density feed × dry type × probiotics; TRT3, high nutrition density feed × wet type × none; TRT4, high nutrition density feed × wet type × probiotics; TRT5, low nutrition density feed × dry type × none; TRT6, low nutrition density feed × dry type × probiotics; TRT7, low nutrition density feed × wet type × none; TRT8, low nutrition density feed × wet type × probiotics.

2) p<0.05 was considered statistically significant, whereas p<0.10 was considered a tendency.

**Table 4:** Effects of feeding program on fecal score in weaning pigs

Items	TRT11)	TRT21)	TRT31)	TRT41)	TRT51)	TRT61)	TRT71)	TRT81)	SEM	p-value2)						
		
	High nutrition density				Low nutrition density					Probiotics	Feed type	Nutrition density	Probiotics × feed type	Feed type × nutrition density	Probiotics × nutrition density	Interaction
	
	Dry type		Wet type		Dry type		Wet type									
	
	NC	Probiotics	NC	Probiotics	NC	Probiotics	NC	Probiotics								
Fecal score3)																
0–5 d	2.5	2.9	2.7	2.8	2.9	2.8	2.9	3.0	0.09	0.039	0.229	0.099	0.624	1.000	0.063	0.229
5–15 d	3.1	2.9	2.9	3.0	3.1	3.0	3.0	3.0	0.06	0.476	0.290	0.476	0.720	0.021	0.476	0.290
15–25 d	3.2	3.0	3.0	3.2	3.3	3.0	3.0	3.1	0.08	0.650	0.184	0.650	0.650	0.003	0.880	0.650

SEM, pooled Standard error of means; NC, negative control.

1) TRT1, high nutrition density feed × dry type × none; TRT2, high nutrition density feed × dry type × probiotics; TRT3, high nutrition density feed × wet type × none; TRT4, high nutrition density feed × wet type × probiotics; TRT5, low nutrition density feed × dry type × none; TRT6, low nutrition density feed × dry type × probiotics; TRT7, low nutrition density feed × wet type × none; TRT8, low nutrition density feed × wet type × probiotics.

2) p<0.05 was considered statistically significant, whereas p<0.10 was considered a tendency.

3) Fecal scores were determined using the following fecal scoring system: 1 hard, dry pellet; 2 firm, formed stool; 3 soft, moist stool that retains shape; 4 soft, unformed stool that assumes shape of container; 5 watery liquid that can be poured.
